# Rare Germline Variants in *CDKN2A*‐Negative Children and Adolescents With Cutaneous Melanoma

**DOI:** 10.1111/pcmr.70079

**Published:** 2026-02-11

**Authors:** Peter A. Johansson, Jane M. Palmer, Linh T. Bui‐Raborn, Mai Xu, Kristine M. Jones, Herbert Higson, Jia Liu, Kelly M. Brooks, Antonia L. Pritchard, Nicholas K. Hayward, Kevin M. Brown

**Affiliations:** ^1^ Oncogenomics Group QIMR Berghofer Brisbane Queensland Australia; ^2^ School of Biomedical Sciences University of Queensland Brisbane Queensland Australia; ^3^ Division of Cancer Epidemiology and Genetics, National Cancer Institute National Institutes of Health Bethesda Maryland USA; ^4^ Cancer Genomics Research Laboratory, Leidos Biomedical Research Frederick National Laboratory for Cancer Research Frederick Maryland USA; ^5^ Genetics and Immunology Department University of the Highlands and Islands Inverness UK

**Keywords:** adolescent, cutaneous melanoma, genetic risk, germline mutation, predisposition

## Abstract

Cutaneous melanoma is a complex disease influenced by both environmental and genetic factors. Inherited susceptibility plays a significant role, involving a combination of high‐, intermediate‐ and low‐penetrance genes. Melanoma in children and adolescents has been speculated to have a stronger genetic component due to the early onset. This study investigates germline variants in early‐onset melanoma through exome sequencing of 154 patients in Australia diagnosed with cutaneous melanoma before the age of 20. Potentially pathogenic variants in shelterin complex genes were identified in 3% of the cases, consistent with a role for telomere dysregulation in early‐onset melanoma. *MC1R* R‐alleles, associated with red hair, fair skin and increased melanoma risk, were less frequent than in adult cases (0.46 vs. 0.64). Pathogenic germline variants in pigmentation genes linked to albinism were identified in 11 individuals (7%), including three truncating variants in *PMEL*, reinforcing the role of pigmentation pathways in cutaneous melanoma susceptibility. Two patients carried mutations in *MBD4*, suggesting it may contribute to early‐onset disease. The high frequency of rare variants in high‐ or intermediate‐risk genes highlights the importance of including such genes in genetic tests, as they may have implications for future risk in the adolescent patients and their at‐risk relatives.

## Key Points

- This study examined exonic variants in 154 individuals diagnosed with cutaneous melanoma in Australia before the age of 20 years.
- *MC1R* R‐alleles, which are associated with melanoma risk in adults, were less frequent in this early‐onset cohort.
- Several variants were identified in shelterin complex genes involved in telomere biology.
- Eleven participants carried variants in pigmentation genes associated with albinism, highlighting the importance of pigmentation pathways in early‐onset melanoma susceptibility.
- Two individuals carried truncating *MBD4* variants, suggesting that monoallelic loss of *MBD4* may contribute to early‐onset cutaneous melanoma.

## Graphical abstract

Individuals with childhood and adolescent melanoma carry inherited mutations in shelterin complex genes, pigmentation genes and MBD4, but have fewer *MC1R* R‐alleles than adults with melanoma. Created with BioRender.com.

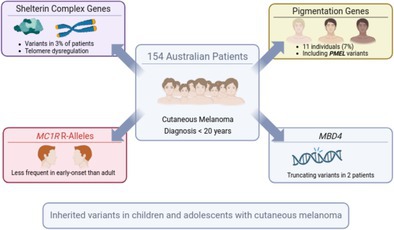

Cutaneous melanoma (CM) is a complex disease influenced by both environmental and genetic factors. Inherited susceptibility plays a significant role, with rare high‐penetrance variants conferring a substantial increase in risk. The most well‐established of the susceptibility genes is in the tumour suppressor *CDKN2A*, which encodes the p16^INK4A^ and p14^ARF^ proteins involved in cell cycle regulation (Hussussian et al. 1994). Mutations in *CDK4*, another cell cycle regulator, are also strongly associated with familial CM, though they are much rarer (Zuo et al. 1996). More recently, genes involved in telomere maintenance, particularly components of the shelterin complex such as *POT1*, *TERF2IP* and *ACD*, as well as mutations to the promoter of the telomerase gene (*TERT*) itself (Horn et al. 2013), have been implicated in CM‐prone families (Aoude, Pritchard, et al. 2015; He et al. 2020; Jensen et al. 2023; Robles‐Espinoza et al. 2014; Shi et al. 2014). In addition, germline mutations in *BAP1* have been linked to a tumour predisposition syndrome that includes cutaneous and uveal melanoma (Abdel‐Rahman et al. 2011; Harbour et al. 2010; Wadt et al. 2012; Walpole et al. 2018). Beyond these high‐penetrance genes, moderate‐penetrance variants also contribute to CM susceptibility. One of the most well‐characterised is *MC1R*, with several common variants associated with red hair, fair skin and CM risk (Landi et al. 2020; Palmer et al. 2000; Valverde et al. 1995). Other examples include DNA repair genes such as *ATM* (Dalmasso et al. 2021), and the *MITF* p.Glu318Lys variant, which confers intermediate risk by affecting the transcription of melanocyte genes (Bertolotto et al. 2011; Yokoyama et al. 2011). Genes involved in oculocutaneous albinism, including *TYR*, *OCA2*, *TYRP1* and *SLC45A2*, have also been linked with intermediate CM risk (Landi et al. 2020; Nathan et al. 2019).

Recently, we analysed the exome from 50 paediatric CM patients (aged younger than 15 years at time of onset of CM) to investigate the contribution of rare germline variants to early‐onset CM (Johansson et al. 2023). Here, we complement that prior work by conducting a study of 154 patients diagnosed with CM in Australia before the age of 20, using exome sequencing to assess both known and candidate CM susceptibility genes (Table S1). Patients from the previous cohort were excluded from this study. With the goal of finding novel or rarer predisposing variant, childhood/adolescent cases who had previously screened for *CDKN2A* mutations and were found to carry a known germline pathogenic variant were excluded from this study, as were cases who belonged to families known to carry a *CDKN2A* mutation, irrespective of whether the individual had been sequenced for that gene. This exclusion may lead to an underestimation of the overall burden of inherited risk contributing to early‐onset CM.

Blood or saliva samples were obtained with informed written consent from the donor or their parent/guardian following approval from the Human Research Ethics Committee of QIMR Berghofer. Blood DNA was extracted using standard salting‐out methods and saliva DNA was collected and extracted using Oragene DNA kits (OG‐500, DNA Genotek, Ottawa, Canada) according to the manufacturer's instructions. Whole‐exome sequencing was performed by the NCI Division of Cancer Research Cancer Genomics Research Laboratory (using the KAPA HyperExome kit; sequencing on an Illumina NovaSeq 6000) (Frederick, MD, USA) or Macrogen (using Illumina SureSelect V7‐Post) (Seoul, South Korea). Sequence reads were mapped to the HumanG1Kv37 assembly using BWA (Li and Durbin 2010), sorted with samtools (Li et al. 2009), and duplicate reads were marked with Picard. Alignments were fine‐tuned by realigning against common variants with GATK IndelRealigner, base qualities calibrated with GATK BaseRecalibrator, variants were identified using GATK HaplotypeCaller (McKenna et al. 2010), validated with an in‐house tool, and annotated using the Variant Effector Predictor (VEP) (McLaren et al. 2016), gnomAD version 4.1 (Chen et al. 2024), AlphaMissense (Cheng et al. 2023), ClinVar categories (downloaded 8 December 2024) and PhyloP scores (Pollard et al. 2010) retrieved through the UCSC Genome Browser (Perez et al. 2025). To identify high‐quality variants we required a phred‐scaled likelihood for the wildtype to be at least 70 and we removed variants in segmentally duplicated regions (Bailey et al. 2002), variants showing mapping quality bias (MQRank > 4), or position bias (ReadPosRankSum > 4) between variant and wildtype reads. Non‐synonymous *MC1R* variants were categorised into *R* (associated with red hair) and *r* alleles as described previously (Robles‐Espinoza et al. 2016). To identify pathogenic variants, we focused on those with a gnomAD Non‐Finnish European allele frequency less than 0.005 and classified as high‐ or moderate‐impact by VEP, that is, variants predicted to truncate the protein or alter the amino acid sequence. Variants classified as benign or likely benign in ClinVar and missense variants not predicted to be pathogenic by AlphaMissense were excluded, unless classified as pathogenic or likely pathogenic in ClinVar.

The 154 patients were recruited from 1987 through 2023 and the median age at diagnosis was 17 years (range 5–19). We first assessed well‐established high‐penetrance melanoma susceptibility genes (Table S2). Five individuals (3%) carried variants meeting the filtering criteria described above in genes encoding components of the shelterin complex. These included two protein‐truncating variants, *POT1* p.(Gln94ArgfsTer13) and *TERF2IP* p.(Phe21SerfsTer2), and three missense variants predicted to be pathogenic by AlphaMissense: *POT1* p.(Lys33Glu), *TERF2IP* p.(Gly60Arg) and *TERF1* p.(Cys126Arg) (Table 1). The missense *POT1* p.(Lys33Glu) lies within the OB1 domain and is specifically known to bind to the telomeric single‐stranded DNA. Its classification as pathogenic by AlphaMissense, along with the saturation genome editing data, indicating a depletion of function when present (Martin et al. 2025) suggests a significant structural and functional impact on the protein, further supported by complete conservation of the affected amino acid across 100 vertebrate species (PhyloP score 5.4). The bases altered by the *TERF2IP* (p.Gly60) and *TERF1* (p.Cys126) variants are likewise highly conserved (PhyloP score 5.1 and 4.8, respectively), which, together with their classification as pathogenic by AlphaMissense, support a potential impact on protein function. Pathogenic *TERF1* variants have previously been identified in a large screen of patients with sarcoma, including one individual with familial melanoma (Ballinger et al. 2023). We did not observe potentially pathogenic variants in *BAP1*, *CDK4*, *ACD* or *TINF2*, nor did we detect any pathogenic *TERT* promoter mutations.

**TABLE 1:** Selected variants in genes associated with melanoma and other cancers.

Sample ID	Gene	Change^a^	Clinvar^b^	AlphaMissense	PhyloP	GnomAD VAF^c^
MELA_1052	MBD4	p.(Glu314ArgfsTer13)	CC	—		4.0 × 10^−4^
MELA_1084	MBD4	p.(TYR539TER)	—	—	5.2	0
MELA_0974	OCA2	p.(Ile634Asn)	LPV	Pathogenic	8.1	1.6 × 10^−4^
MELA_1108^d^	OCA2	p.(Asn489Asp)	PV/LPV	Pathogenic	5.6	7.1 × 10^−4^
MELA_1020	OCA2	p.(Pro743Leu)	PV/LPV	Pathogenic	8.6	1.8 × 10^−4^
MELA_1009	PMEL	p.(Leu68SerfsTer10)	—	—		0
MELA_1013	PMEL	p.(Val81SerfsTer45)	—	—		1.3 × 10^−4^
MELA_1082	PMEL	p.(Tyr49Ter)	—	—	0.6	4.4 × 10^−5^
MELA_1109	POT1	p.(Lys33Glu)	VUS	Pathogenic	5.4	0
MELA_0996	POT1	p.(Gln94ArgfsTer13)	PV/LPV	—		0
MELA_1024	TERF1	p.(Cys126Arg)	—	Pathogenic	4.8	0
MELA_1094	TERF2IP	p.(Phe21SerfsTer2)	—	—		0
MELA_1043	TERF2IP	p.(Gly60Arg)	CC	Pathogenic	5.1	0
MELA_1082	TP53	p.(Lys120Asn)	—	Pathogenic	0.8	0
MELA_1043	TYR	p.(Thr373Lys)	PV	Pathogenic	5.5	9.7 × 10^−4^
MELA_1111^d^	TYR	p.(Thr373Lys)	PV	Pathogenic	5.5	9.7 × 10^−4^
MELA_1046	TYR	p.(Val275Phe)	PV/LPV	Benign	−0.1	3.7 × 10^−4^
MELA_0970	TYRP1	c.913+2T>G	PV/LPV	—	7.7	0
MELA_1099	TYRP1	p.(Leu379Phe)	—	Pathogenic	5.4	0

*Note:* High and moderate impact variants not classified as benign in ClinVar were considered; moderate impact variants were required to be classified as pathogenic in ClinVar or predicted pathogenic by AlphaMissense. See Tables S2 and S3 for more details.

Abbreviations: CC, conflicting classifications; LPV, likely pathogenic variant; PV, pathogenic variant.

^a^ The variant's predicted change in the protein encoded by the MANE select transcript.

^b^ The aggregate classification in the ClinVar database.

^c^ The variant allele frequency in the non‐Finnish European population in gnomAD v4.1.

^d^ Individual was included in Nathan et al. (2019).

We next assessed moderate‐penetrance susceptibility genes, including *MITF* p.Glu318Lys, *ATM* and well‐established albinism and pigmentation genes. The known *MITF* p.Glu318Lys (Yokoyama et al. 2011) variant was identified in two individuals (1.3%), a frequency that lies between that reported in the general population (0.71%, Fisher's exact test, *p =* 0.3) and in adult CM patients (2.1%; Fisher's exact test, *p* = 0.74) (Potrony et al. 2016). The cohort size is, however, insufficient to draw statistically meaningful conclusions. We did not identify other potentially pathogenic *MITF* variants. Four variants were found in *ATM*, including a frameshifting variant p.(Ser1905IlefsTer25), splice‐site variant c.7630‐1G>A, and two missense variants p.(Pro292Leu) and p.(Tyr1442His). In terms of pigmentation genes, on average patients carried 0.46 *MC1R R* alleles. This is significantly lower than the 0.64 reported previously in a cohort of 303 adult CM patients studied as part of the Australian Melanoma Genome Project (Fisher's exact test, *p* = 0.007) and comparable to that observed in uveal melanoma (UM) patients in the same study (0.48) (Newell et al. 2022). As there is no significant association between *MC1R* genotypes and UM risk (Hearle et al. 2003; Mies et al. 2025) these data suggest that while *R* alleles are linked to an increased risk of CM (Bishop et al. 2009; Landi et al. 2020; Raimondi et al. 2008), they are not a major risk factor for childhood/adolescent CM. In our previous study of childhood CM cases (Johansson et al. 2023), the frequency of *R* alleles was not significantly lower than in adult cases (mean 0.56, 95% confidence interval (CI) 0.39–0.76), possibly due to the limited sample size and wide confidence interval. When combining the two childhood/adolescent cohorts the *R* allele frequency was 0.49 (95% CI 0.40–0.59), still significantly lower than in the Australian adult CM cases (odds ratio (OR) 0.69, Fisher's exact test, *p* = 0.014) (Newell et al. 2022). In comparison, in a large multicentre study of 233 young cases (most cases from Italy, the Netherlands, Spain, France and Sweden), *R* alleles were identified as a significant risk factor in both childhood/adolescent and adult cases, and there was no substantial difference in *R* allele frequency between the two groups (Pellegrini et al. 2019). The reason for this difference is not clear. It may reflect a variation in the prevalence of *R* alleles across different populations, as the majority of Australian patients are of British ancestry, whereas the multicentre study included a more diverse sample with greater representation from Southern Europe. Higher levels of ultraviolet radiation (UVR) in Australia than in Europe may also contribute to the difference, as well as differences in sun protection behaviour between different countries, age groups and skin type. Epidemiological studies are needed to address these possibilities.

Additionally, eight pathogenic variants were found in oculocutaneous albinism (OCA) genes, affecting *OCA2* (*n* = 3), *TYR* (*n* = 3) and *TYRP1* (*n* = 2) (Table 1). We also identified three individuals with germline protein‐truncating variants (PTVs) in *PMEL*, a gene encoding a key structural component of the melanosome, which is essential for melanin synthesis, storage and transport. *PMEL* has previously been linked to cancer through recurrent somatic mutations (Zhang et al. 2015). The mouse homolog of *PMEL* is associated with a coat colour phenotype (silver) (Kwon et al. 1995), and biallelic loss of *PMEL* has recently been linked to oculocutaneous albinism (AlAbdi et al. 2023). These findings suggest that monoallelic loss of *PMEL* contributes to susceptibility to CM, analogous to the role of other OCA‐associated genes such as *TYR*, *OCA2*, *TYRP1* and *SLC45A2*, in which biallelic loss causes oculocutaneous albinism and heterozygous variants have been associated with increased CM risk (Landi et al. 2020; Nathan et al. 2019).

Four individuals carried variants in genes previously linked to non‐sun‐exposed melanoma subtypes. Two individuals harboured PTVs in *MBD4* p.(Glu314ArgfsTer13) and p.(Tyr539Ter), a gene associated with an increased risk of uveal melanoma (Derrien et al. 2021; Villy et al. 2024). We also previously reported a single case of an *MBD4* PTV in a cohort of 50 paediatric CM patients (Johansson et al. 2023), bringing the total to three carriers (1.5%), suggesting that *MBD4* may predispose to early‐onset CM. Another individual carried a missense variant in *PLCB4* (p.Ser342Leu), predicted to be pathogenic by AlphaMissense. However, this variant differs from the well‐characterised gain‐of‐function *PLCB4* mutation (p.Asp630), which is often somatically acquired in uveal melanoma tumours and activates the Gq/11‐dependent signalling pathway (Johansson et al. 2016). Finally, one individual carried a missense variant in *WRN*, p.(Lys483Asn), which is predicted to be pathogenic by AlphaMissense. *WRN* is primarily associated with Werner syndrome but has also been linked to acral melanoma (Goto et al. 1996; Newell et al. 2022). The carrier reported here had no record of acral melanoma.

Lastly, we examined genes linked with other cancers (Table S1), restricting our analysis to rare variants with a gnomAD Non‐Finnish European allele frequency less than 5 × 10^−4^ (Table S3). One individual carried a *TP53* p.(Lys120Asn) variant, a missense alteration predicted to be pathogenic by AlphaMissense. The variant is extremely rare in the germline, having not been observed in a dataset of 800,000 individuals (gnomAD 4.1) and is therefore absent from clinical variant databases such as ClinVar. Somatically, it has been classified as a recurrent hotspot (Chang et al. 2016), including in databases such as cBioPortal. It was also included in a systematic functional screen, in which it received a relative fitness score of −0.135, intermediate between the scores of silent variants (mean −2.50) and PTVs (mean 0.42), suggesting a potential intermediate impact on the p53 function (Kotler et al. 2018).

Four individuals collectively carried variants in *POLE* p.(Cys501Ter), p.(Pro282Ser), p.(Lys284Glu), or *POLD1* p.(Leu983ArgfsTer65), two genes encoding proteins involved in DNA proofreading. Both missense variants occurred in the exonuclease domain (amino acids 268–471), consistent with a likely impact on proofreading capability. Loss of function of these proteins has primarily been linked to colon cancer but has also been reported in other cancers, including cutaneous melanoma (Aoude, Heitzer, et al. 2015; Mur et al. 2020). Apart from the four variants in *ATM*, another 13 variants were identified in genes involved in homologous recombination repair, including five PTVs in *BRIP1*, *BARD1*, *CHEK2*, *FANCA* and *FANCF*. Three individuals carried pathogenic variants in *XPA*, *XPC* and *ERCC1*, genes involved in nucleotide excision repair. Biallelic loss of these genes causes xeroderma pigmentosum, a rare recessive disorder characterised by extreme sensitivity to UV radiation. We recently reported two patients affected by both uveal and cutaneous melanoma who carried pathogenic variants in XP genes (Johansson et al. 2025). The presence of these three variants in this cohort further supports the hypothesis that monoallelic loss of these genes increases UV sensitivity and elevates CM risk.

One individual carried a *DNMT3A* p.(Arg882Cys) variant affecting a well‐characterised hotspot for somatic mutations in myeloid leukaemias (Ley et al. 2010). This mutation is a known early event in disease development, frequently observed in clonal haematopoiesis and myeloid malignancies. In contrast, germline mutations in *DNMT3A* cause Tatton–Brown–Rahman syndrome, a development disorder associated with overgrowth and neurodevelopment delay (Tatton‐Brown et al. 2014). Notably, the carrier did not exhibit features of this syndrome but was diagnosed with a haematological malignancy at age 73, approximately a decade after donating blood for this study, suggesting that the *DNMT3A* variant had likely been acquired somatically and therefore unrelated to her early‐onset melanoma. We observed another five pathogenic variants in genes linked with haematological malignancies, including *ROS1* p (Asp839Glu), *JAK3* p.(Val946Leu) and three PTVs in *RUNX1* (Table S3), also possibly acquired somatically.

Several individuals carried more than one potentially pathogenic variant. For example, MELA_1082 and MELA_1043 carried variants in *PMEL*/*TP53* and *TYR*/*TERF2IP*, respectively (Table 1). Others carried combinations such as *ATM/MITF*, *CHEK2/POLD1*, *POT1/POLE*, *ATM/TERF1*, and *OCA2/FANCF* (Tables S2 and S3). These findings highlight several observations: first, they support the notion that many of these variants have intermediate penetrance, consistent with previous suggestions for OCA genes (Nathan et al. 2019) and population‐based estimates of *POT1* variant penetrance (OR = 2.11) (Simonin‐Wilmer et al. 2022). Second, there is a trend where variants in pigmentation genes such as *PMEL*, *MITF* and *OCA2* co‐occurred with variants in DNA repair genes such as *TP53*, *ATM*, *XPA*, *POLE* and *FANCF*. This likely reflects the multilayered protection melanocytes rely on against mutagenic UVR with melanin acting as a physical shield, followed by repair mechanisms that correct UVR‐induced DNA damage. Similarly, the co‐occurrence of variants in shelterin genes (*TERF2IP*, *POT1* and *TERF1*) with pigmentation or DNA repair genes reminds us that while telomere maintenance is required for limitless replication, it is not sufficient for tumorigenesis. Aside from germline variants, a melanocyte must acquire multiple cancer‐enabling mutations somatically to become malignant (Hanahan and Weinberg 2011). In contrast to adult cancer, where such acquired changes may be accumulated over extended periods of time, in childhood/adolescent cancers there may be greater reliance on inherited germline variants.

In conclusion, five individuals (3%) carried pathogenic variants in *POT1*, *TERF1* or *TERF2IP*, supporting a significant role for shelterin complex genes in early‐onset CM. A lower‐than‐expected frequency of *MC1R* R‐alleles, typically associated with fair skin and melanoma risk, was observed. Instead, 11 individuals (7%) carried pathogenic germline variants in pigmentation genes linked to oculocutaneous albinism, including three with protein‐truncating variants in *PMEL*, reinforcing the role of pigmentation genes in CM predisposition. Truncating variants in *MBD4* were identified in two individuals; together with a previously reported paediatric case, these findings are consistent with a potential role for *MBD4* in early‐onset CM. The high frequency of rare variants associated with high or intermediate melanoma risk underscores the importance of incorporating such variants into genetic risk assessments. As our understanding of melanoma genetics evolves, comprehensive risk profiles will be crucial for interpreting genetic tests and guiding long‐term cancer surveillance in both early‐onset CM patients and their at‐risk relatives.

## Author Contributions

Conceptualisation and funding acquisition: N.K.H.; data curation: P.A.J., J.M.P., L.T.B.‐R., A.L.P.; formal analysis, software and writing – original draft preparation: P.A.J.; methodology: P.A.J., A.L.P.; investigation: L.T.B.‐R., M.X., K.M.J., H.H., J.L., K.M.B., resources: J.M.P., N.K.H., Kev.M.B, writing – review and editing: P.A.J., J.M.P., L.T.B.‐R., M.X., K.M.J., Kel.M.B., A.L.P., N.K.H. and Kev.M.B.

## Funding

This work was supported by the National Health and Medical Research Council (APP1195581), National Cancer Institute, Buck Off Melanoma.

## Conflicts of Interest

The authors declare no conflicts of interest.

## Supporting information


**Table S1:** List of cancer genes.
**Table S2:** Variants identified in cutaneous melanoma susceptibility genes.
**Table S3:** Variants identified in other cancer genes.

## Data Availability

Data have been deposited at the European Genome‐phenome Archive (EGA), which is hosted by the EBI and the CRG, under accession number EGAS50000001311, https://ega‐archive.org/studies/EGAS50000001311.
